# Six-Year BDS-2 Broadcast Navigation Message Analysis from 2013 to 2018: Availability, Anomaly, and SIS UREs Assessment

**DOI:** 10.3390/s19122767

**Published:** 2019-06-20

**Authors:** Chenhao Ouyang, Junbo Shi, Yuru Shen, Lihong Li

**Affiliations:** 1 School of Geodesy and Geomatics, Wuhan University, Wuhan 430079, China; ouyangchenhao@whu.edu.cn (C.O.); shenyuru@hotmail.com (Y.S.); sggllh@163.com (L.L.); 2 Key Laboratory of Precise Engineering and Industry Surveying, National Administration of Surveying, Mapping and Geoinformation, Wuhan 430079, China

**Keywords:** BDS-2, anomaly detection, global-average URE, orbit-only URE, worst-case URE

## Abstract

The second-generation of the Beidou Navigation Satellite System (BDS-2) has been officially providing positioning, navigation, and timing (PNT) services within the Asia–Pacific region for six years, starting from 2013. A comprehensive analysis of BDS-2 satellite broadcast navigation message performance during the past six years is highly demanded, not only for the regional service but also for the global service announced in December 2018. Therefore, this study focuses on the performance assessment of six-year BDS-2 broadcast navigation messages from 2013 to 2018 in three aspects: Message availability, anomaly detection, and signal-in-space user range errors (SIS UREs). Firstly, our results, based on International GNSS service (IGS) Multi-GNSS Experiment (MGEX) navigation files, indicate that the BDS-2 Geosynchronous Earth Orbit (GEO) and Inclined Geosynchronous Satellite Orbit (IGSO) satellites have >98.51% broadcast navigation message availability, and the Medium Earth Orbit (MEO) satellites has a ~90.03% availability. Secondly, the comparison between broadcast navigation messages and IGS precise products reveals that the User Range Accuracy Index (URAI) contained in the broadcast message could not reflect satellite performance correctly. Another satellite status indicator, space vehicle (SV) health, can only partially detect a satellite anomaly. The anomaly detection result using IGS precise products for reference shows 20241 anomalies out of 651038 broadcast navigation messages within six years. Finally, compared with the IGSO and MEO satellites, the orbit qualities of GEO satellites are significantly worse due to their large along-track orbit error. The clock performance of all satellites are at the comparable level. The satellite orbit type (GEO/IGSO/MEO) does not impact the orbit-only URE, global-average URE, and worst-case URE.

## 1. Introduction

The second-generation of the Beidou Navigation Satellite System (BDS-2) has been providing real-time positioning, navigation, and timing (PNT) services since 27 December 2012. As indispensable information for BDS-2 PNT applications, the satellite orbit and clock correction can be retrived from broadcast navigation messages [1,2]. The existence of any imperfect navigation message or frequent satellite orbit/clock adjustment would directly deteriote the performance of BDS-2 broadcast orbit and clock correction [3], which would then bring potential barriers for widespread BDS-2 application. Therefore, it is of great importance to assess the availability, anomalies, and accuracy of BDS-2 broadcast navigation messages.

Previous studies mainly focused on BDS-2 satellite anomaly detection and accuracy evaluation through analyses of BDS-2 broadcast navigation messages. In order to maintain their geosynchronous status, the BDS-2 Geosynchronous Earth Orbit (GEO) and Inclined Geosynchronous Satellite Orbit (IGSO) satellites would be frequently maneuvered by ground control segments [4]. During the maneuver period, the solar radiation model, phase wind-up model, and antenna phase center offset (PCO) model are different from their nominal statuses [5]. A large orbit error would be induced due to an inaccurate force model, which would certainly degrade the user positioning performance. In addition, some other factors, such as the satellite inbound/outbound transmissions, would cause broadcast orbit and clock anomalies [6,7]. By computing orbit and clock differences between adjacent broadcast navigation messages, an empirical model was developed to detect satellite anomalies by Jan [8,9]. One-month BDS-2 broadcast navigation messages in 2013 were utilized to generate the empirical model. Similar to Jan’s method, Ye et al. [10] proposed another empirical model by calculating mutual differences of orbit and clock correction at multiple epochs. By taking the proposed method into the precise orbit determination (POD), the authors correctly detected the C03/C08 maneuver on 5/9 January, 2015. Comparable POD accuracy was obtained for the maneuvered satellites.

Another category of BDS-2 satellite anomaly detection based on both broadcast navigation messages and satellite observations has been investigated in recent years. A large error in the broadcast navigation message could enlarge the satellite observation residual. This principle can be applied to satellite anomaly detection by static stations with known positions. The anomaly detection method proposed by Huang et al. [11] was able to locate the exact start and end time of a BDS-2 satellite maneuver. With data collected by one of the International GNSS Service (IGS) Multi-GNSS Experiment (MGEX) stations in 2016, the method was proven effective with a total of 58 GEO and 12 IGSO maneuvers detected. An improved method was proposed afterward by Huang et al. [12] in 2018. With the optimized method, 32/13/1 orbital maneuvers and 102/13/12 non-maneuvering anomalies are detected for the GEO/IGSO/Medium Earth Orbit (MEO) satellites from day-of-year (DOY) 1 to 300 in 2017. As opposed to the single station experiment in Huang’s contribution, Jiang et al. [13] applied 13 MGEX stations over the Asia–Pacific region to detect BDS-2 satellite anomalies. A total of 35 BDS-2 anomalies were detected from DOY 305 to 361 in 2015.

In addition to the satellite anomaly detection, the orbit accuracy and clock precision of BDS-2 broadcast navigation messages were also intensively studied. Chen et al. [14] assessed BDS-2 broadcast navigation messages from 2013 to 2014 and reported 3.0 and 5.0 m orbit accuracy for IGSO/MEO and GEO, respectively. Better than 8 ns clock precision was also obtained. The influence of broadcast navigation message errors is actually projected onto the line-of-sight (LOS) direction for users. A signal-in-space user range error (SIS URE) is applied for a comprehensive BDS performance assessment. With one-year broadcast navigation messages in 2013, Montenbruck et al. [15] evaluated the BDS-2 broadcast navigation message SIS URE and concluded that they had a 1.5 m global-average SIS URE. A comparison between BDS-2 and GPS/GLONASS/GALILEO/QZSS showed that the BDS-2 SIS URE was comparable with GLONASS and GALILEO but slightly worse than GPS and QZSS. With BDS-2 broadcast navigation messages in August 2016, 1.87 m and 2.17 m SIS UREs—considering both orbit and clock errors—were reported for the IGSO/MEO and GEO satellites, respectively [16]. Wu et al. [17] analyzed the BDS-2 SIS UREs from 2013 to 2016. Their results indicated that the User Range Accuracy Index (URAI) indicator cannot properly reflect the satellite usage status and would mislead advanced receiver autonomous integrity monitoring (ARAIM) users. Wang et al. [18] illustrated the BDS-2 SIS URE’s influence on ARAIM false alert Probability (Pfa) with broadcast navigation messages from 2013 to 2017. Their result showed that the 2.4 m user range accuracy (URA) standard deviation can completely overbound SIS UREs for all BDS satellites, and the corresponding Pfa was 2 × 10^−5^.

On the one hand, a comprehensive analysis of BDS-2 satellite broadcast navigation message performance during the past six years is meaningful for regional PNT users. On the other hand, these BDS-2 satellites will start to provide global service as a critical component of the BDS-3 preliminary system from December 2018. Therefore, it is of great value and importance to assess the BDS-2 satellite broadcast navigation message performance, which is the focus of our study. This paper concerns the six-year BDS-2 satellite broadcast navigation message performance evaluation from the perspectives of broadcast message availability, anomaly detection, and SIS URE calculation. Section 2 describes the background of the current BDS-2 consellation. In Section 3, an analyzing flowchart of BDS-2 broadcast navigation messages is introduced. Six-year analyzing results are presented in Section 4. Conclusions are finally reported in Section 5.

## 2. BDS-2 Satellite Constellation Development

**Figure 1:**
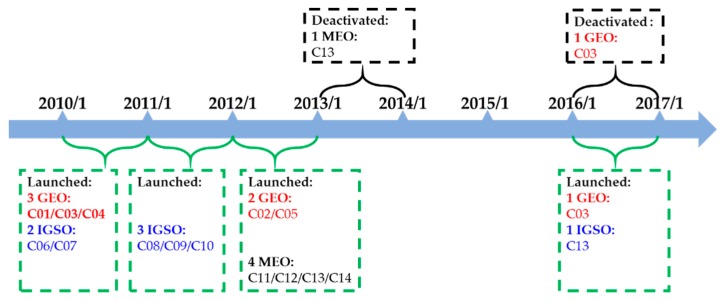
The second-generation of the Beidou Navigation Satellite System (BDS-2) satellite launching and deactivation status.

BDS-2 has been providing Asia–Pacific regional service since 27 December 2012. Currently, there are 14 operational BDS-2 satellites, including five GEO, six IGSO, and three MEO, with their launching and deactivation status shown in Figure 1. During the six-year regional service provision period, C03 was updated by a new satellite on September 29, 2018. Meanwhile, C13 was launched as an MEO satellite from September 19, 2012 to October 21, 2014. After that, C13 was retired from the BDS-2 constellation. Two years later, a new C13 satellite started to provide service as an IGSO satellite [19]. All these satellites contribute to the BDS-2 satellite constellation. Since November 2017, nine MEO, two IGSO, and one GEO satellites have been launched to construct the BDS-3 preliminary system [20].

## 3. Material and Methods

MGEX collects multi-GNSS broadcast navigation messages with globally distributed GNSS receivers. These navigation messages are recorded into daily archives which are available at the IGS website: ftp://cddis.gsfc.nasa.gov/pub/gps/data/campaign/mgex/daily/rinex3/. BDS broadcast navigation messages are subsumed into merged navigation files since DOY 42, 2013. This paper retrieved six-year MGEX archived broadcast navigation message files from 2013 to 2018. Orbit accuracies of MGEX BDS-2 precise products were reported as several decimeters, one decimeter, and centimeters for GEO, IGSO, and MEO satelltes, respectively [21]. As for precise clock products, about 0.8/0.3/0.2 ns precision could be obtained for the GEO, IGSO, and MEO satellites, respectively [22]. As the MGEX precise orbit/clock products are significantly better than the broadcast navigation messages, the BDS-2 precise orbit/clock products generated by Wuhan University, one major IGS MGEX contributor, were selected as the reference for broadcast navigation message performance assessment.

**Figure 2:**
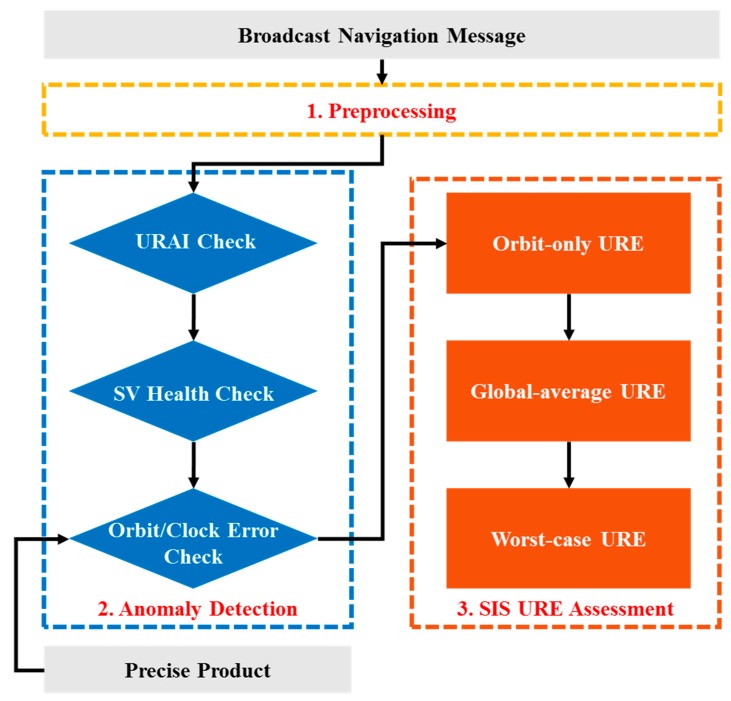
BDS broadcast navigation message analyzing flowchart.

After all necessary datasets were collected, a comprehensive evaluation method was proposed to assess the six-year BDS-2 broadcast navigation messages. Figure 2 shows the evaluation flowchart. Firstly, a preprocessing step was carried out to exclude invalid data. The statistics of broadcast navigation message availability were then provided. Secondly, a three-factor (URAI, space vehicle health flag, and external precise product) anomaly detection step was developed to explore all existing broadcast navigation message anomalies. Finally, a multi-SIS-URE assessment step was performed to investigate the overall BDS-2 orbit/clock performance.

### 3.1. Preprocessing

Invalid BDS broadcast navigation messages under three conditions are excluded from this study:

- Redundant broadcast navigation messages. Multiple message blocks with the same content are recognized as redundant messages.
- Incomplete broadcast navigation messages. Message blocks with blank orbit/clock parameters are recognized as incomplete messages.
- Unevaluable broadcast navigation messages. If the precise orbit/clock references are unavailable, the correspondent broadcast navigation messages are recognized as unevaluable messages.

### 3.2. Anomaly Detection

After removing invalid navigation messages in Section 3.1, abnormal navigation messages should be detected and rejected before the nominal SIS URE assessment in Section 3.3.

• Anomaly detection with the URAI and space vehicle (SV) health flags contained in the broadcast message

According to the BDS-2 Interface Control Document (ICD), two parameters are designed in the broadcast navigation message for satellite anomaly detection: The URAI and SV health. The URAI is an indicator for User Range Accuracy (URA), which is used to describe the satellite signal-in-space accuracy in the unit of meter [23]. The relationship between the URAI and URA can be found in the BDS-2 ICD. A URAI threshold of seven was selected for anomaly detection in this paper. SV health was selected as the satellite status indicator. The SV health indicator “0” means a healthy satellite, whereas “1” means unhealthy. These two indicators can be applicable for real-time anomaly detection.

• Anomaly detection with external precise products references

With precise orbit/clock products as the reference, we can calculate the broadcast orbit/clock errors. The comparison is carried out referring to time of clock (ToC) for every broadcast navigation message. Since both BDS broadcast orbit and precise orbit refer to the satellite antenna mass center [17], no phase center correction is necessary for the orbit comparison. As for the clock assessment, C01 was selected as the reference satellite to eliminate the clock datum difference between broadcast and precise products [24].

Satellites with significantly large orbit/clock errors are considered as abnormal in this paper. IGSO/MEO orbit error thresholds were set as 3/6/6 m in radial/along-track/cross-track directions, respectively. Radial/along-track/cross-track error thresholds of 3/45/6 m were selected for GEO orbits. The clock error threshold was set to 10 m for all satellites [19].

### 3.3. SIS UREs Assessment

After removing invalid and abnormal broadcast navigation messages, six-year BDS nominal SIS UREs were evaluated. Three indicators were adopted for the SIS UREs assessment: Orbit-only URE, global average URE, and worst-case URE [20].

The orbit-only URE is an assessment of the average URE over the entire earth surface. It only considers the orbit error as shown in Equations (1) and (2) with various coefficients for the MEO and IGSO/GEO satellites, respectively

(1) $$Orbit-only\;{\mathrm{URE}}_{MEO}=\sqrt{{\left(0.98R\right)}^{2}+\frac{1}{54}(A^{2}+C^{2})},$$

(2) $$Orbit-only\;{\mathrm{URE}}_{IGSO/GEO}=\sqrt{{\left(0.99R\right)}^{2}+\frac{1}{127}(A^{2}+C^{2})},$$

where R represents the radial component error, A represents the along-track component error, and C represents the cross-track component error.

The global-average URE is another average URE assessment indicator that takes both orbit and clock error into consideration. Similar to the orbit-only URE, various satellite orbital altitudes result in different coefficient as shown in Equations (3) and (4)

(3) $$Global-average{\;\mathrm{URE}}_{MEO}=\sqrt{{\left(0.98R-T\right)}^{2}+\frac{1}{54}(A^{2}+C^{2})}$$

(4) $$Global-average{\;\mathrm{URE}}_{IGSO/GEO}=\sqrt{{\left(0.99R-T\right)}^{2}+\frac{1}{127}(A^{2}+C^{2})}$$

where T represents the satellite clock error.

The worst-case URE is a third assessment criterion for an instantaneous URE. It describes the maximum instantaneous URE for a user in the satellite coverage. Both orbit and clock errors are considered in Equation (5)

(5) $$Worst-case\;\mathrm{URE}=\underset{\left|\theta \right|\leq \gamma }{\max}(R\cos\theta -T+\sqrt{A^{2}+C^{2}}\sin\theta )$$

where θ is the satellite off-nadir angle and γ is the maximum off-nadir angle within the satellite coverage.

## 4. Results

In this section, we first present the six-year BDS-2 broadcast navigation file and precise product availabilities. Then, the anomaly detection results using the URAI, SV health, and the precise product are reported. The BDS-2 nominal SIS UREs are evaluated after eliminating all detected anomalies.

### 4.1. BDS-2 Broadcast Navigation Message and Precise Product Availability

**Figure 3:**
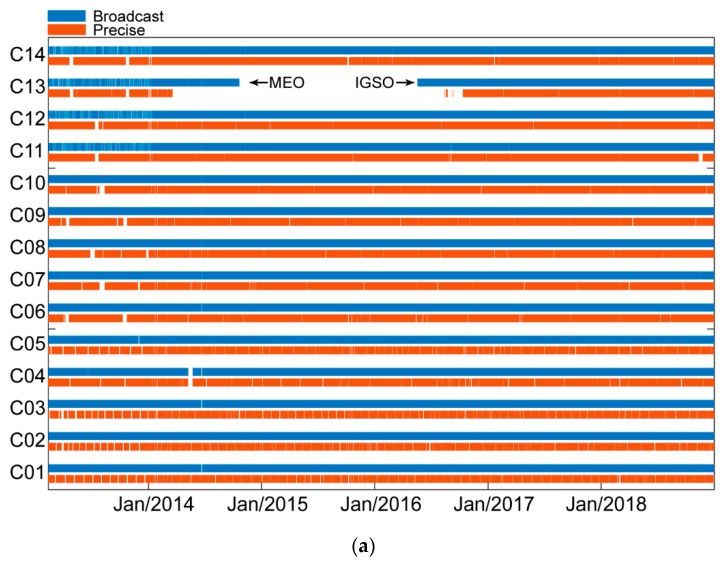

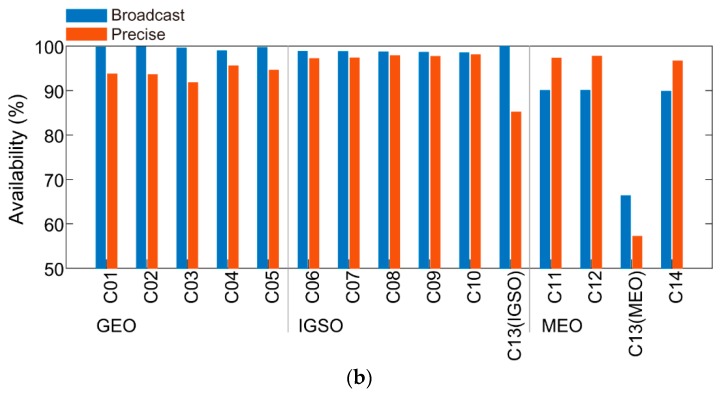
Availability of BDS broadcast navigation message in Multi-GNSS Experiment (MGEX)-merged navigation files and the precise product generated by Wuhan University. (**a**) Six-year series of available BDS-2 broadcast navigation message and the precise product; (**b**) Availability of BDS-2 broadcast navigation message and the precise product.

Figure 3 displays the time series (a) and availability histogram (b) of BDS-2 broadcast navigation messages and the precise product. Blue indicates available broadcast navigation messages, while red indicates the precise product. The availability statistics in Figure 3b are calculated as

(6) $$Availability=\frac{\#\;of\;Available\;Messages}{\#\;of\;Expected\;Messages\;During\;The\;Period\;of\;Service\;}\times 100\%$$

Figure 3a shows that all satellites have been providing consecutive service except for C13. Neither broadcast nor precise product was available for C13 from DOY 294, 2014 to DOY 139, 2016. The data absence was caused by satellite replacement. In the first phase, C13 acted as an MEO satellite. After the C13 retirement, a new IGSO satellite C15 was launched. A pseudo-random noise (PRN) number switch from C15 to C13 was made by the ground control segment after 146 days.

Meanwhile, we can also see from Figure 3 that four MEO satellites (C11–C14) suffered from frequent broadcast navigation message missing in 2013. However, the situation for the precise product was not the same. The BDS precise product availability was more stable than broadcast navigation messages during the same period.

Figure 3b shows the availability comparison between the broadcast and precise product. Discarding the C13 satellite replacement issue, we can see that the broadcast navigation message availability (>98.51%) is greater than that of the precise product (>91.79%) for all GEO and IGSO satellites. As for MEO satellites, the data missing issue in 2013 led to the relatively worse availability of broadcast navigation message (<90.03%) than the precise product (>96.67%).

In the preprocessing stage, 32293-invalid BDS-2 broadcast navigation messages were excluded based on three conditions defined in Section 3.1. A total of 651038 broadcast navigation messages were retained for further analysis.

### 4.2. BDS-2 Anomaly Detection Results

In this section, six-year BDS-2 anomalies are detected. Two satellite indicators contained in the broadcast navigation message, i.e., the URAI and SV health, were used to detect satellite anomalies to see if they can correctly reflect the satellite status. After presenting the URAI and SV health detection results, BDS-2 anomalies detected by comparing broadcast navigation messages with the precise products are presented.

**Figure 4:**
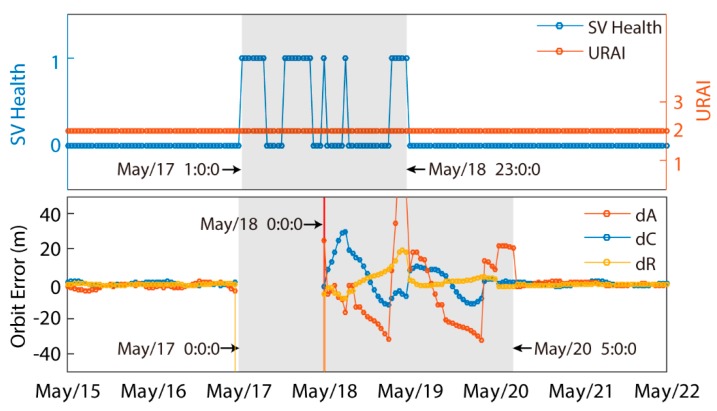
The User Range Accuracy Index (URAI), space vehicle (SV) health (Top) and orbit error (Bottom) time series of C07 broadcast navigation messages from May/15 to May/22 in 2016.

Firstly, a one-week C07 anomaly detection result from 15 May to 22 in 2013 is depicted in Figure 4. An apparent anomaly occurred from May/17 0:0:0 to May/20 5:0:0. We further separate this period into three parts:

(1) May/17 1:0:0–May/18 0:0:0. The URAI in this period retained a value of 2 (3.40–4.85 m URA), whereas SV health changed between 0 (healthy) and 1 (unhealthy). Since no precise product was available for this period, broadcast navigation messages were unevaluable, as illustrated in the Section 3.1.
(2) May/18 0:0:0–May/18 23:0:0. The URAI and SV health patterns were similar to that during the previous period, except that SV health was marked as healthy during most time. However, the precise product was available in this period. The orbit error series showed a poor accuracy worse than 20 m.
(3) May/18 23:0:0–May/20 5:0:0. The URAI in this period still held a value of 2 (3.40–4.85 m URA), while SV health held a value of 0, indicating the satellite was in healthy condition. The orbit error series in this period, however, still showed a worse than 20 m orbit error, which indicates the satellite was not in an applicable condition.

We can conclude from the weekly anomaly detection result that the URAI could not detect any satellite anomalies. SV health can only partially identify the satellite abnormal status. However, the comparison, with respect to the precise product, was able to detect significantly more satellite anomalies than the other two.

Table 1 shows six-year anomaly detection results using the URAI and SV health. The URAI in broadcast navigation messages could not tell any anomalies throughtout the six years. While SV health identified 5261 unhealthy messages, occupying 0.81% of all tested data. The C13 (MEO) was verified to be problematic again with 31.78% broadcast navigation messages marked as unhealthy. The SV health detected unhealthy ratios were 0.09%/0.31%/1.94% for GEO/IGSO/MEO (except for C13), respectively.

Table 2 shows six-year anomaly detection result with reference to the precise products. We can see that a total of 20,241 broadcast navigation messages were detected as anomalies, which occupy 3.11% of all tested data. Among these anomalies, 18,432 messages were detected as orbit anomalies, while only 2011 were detected as clock anomalies. The anomaly ratios are 2.81%/2.92%/4.01% for GEO/IGSO/MEO (without C13), respectively.

**Figure 5:**
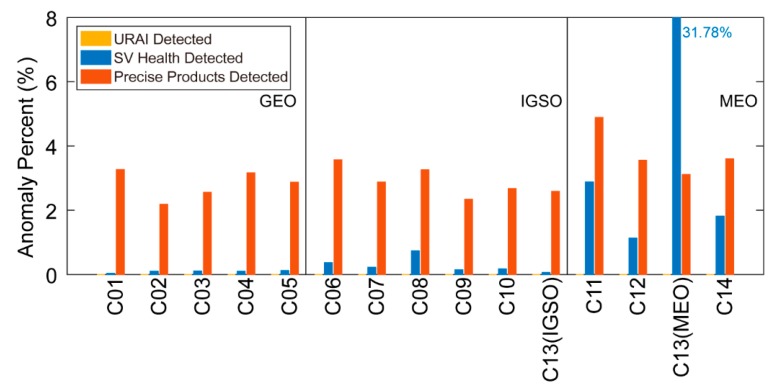
Comparison of the URAI, SV health, and the precise product anomaly detection results.

To further reflect BDS anomalies, Figure 5 illustrates the comparison of anomalies detected by the URAI, SV health, and the precise product. For most satellites, anomalies detected by precise products were more than that by SV health except for C13 (MEO). Though 31.78% messages of C13 (MEO) were marked as unhealthy, only 3.11% were detected as anomalies. The significant difference indicates that, although marked as unhealthy, C13 (MEO) can still broadcast valid orbit/clock messages.

To sum up, three conclusions are summarized in Table 3: (1) The URAI is incapable of detecting any anomalies. (2) The SV health flag can only reflect satellite anomalies partially (5261). (3) With precise products as the reference, 20,241 out of 651,038 broadcast navigation messages were detected as anomalies.

### 4.3. Nominal SIS URE Assessment

**Figure 6:**
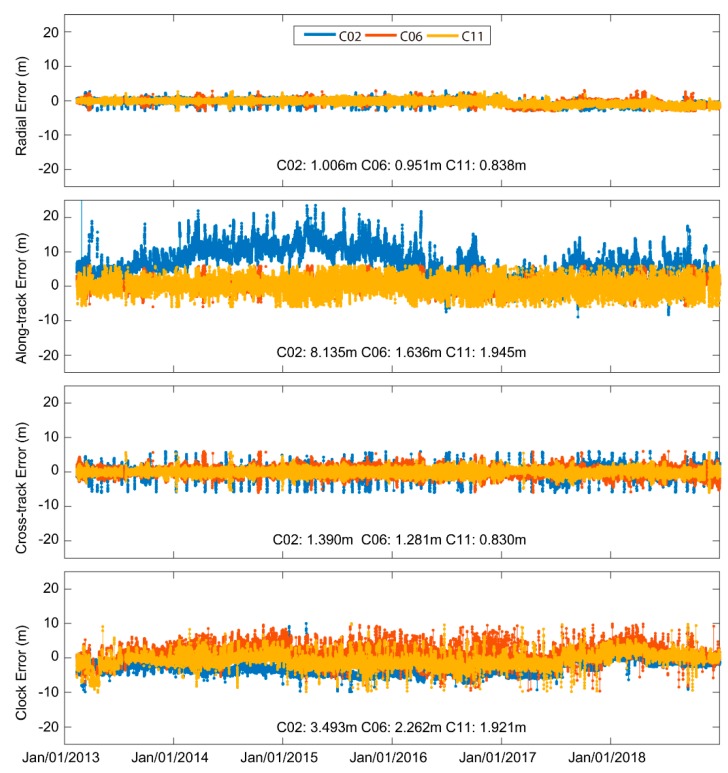
Six-year C02/C03/C11 broadcast navigation message orbit and clock error time series.

After the anomaly detection in Section 3.2, 630,797 (= 651,038−20,241) BDS-2 broadcast navigation messages are retained for nominal SIS URE assessment in this section. First, three satellites C02/C06/C11 are selected as representatives of GEO/IGSO/MEO, respectively. Figure 6 shows a six-year orbit/clock error series of the three selected satellites. The Root Mean Square (RMS) of the radial errors are 1.006/0.951/0.838 m, respectively, which are the best out of the three directions. The RMSs of 8.135/1.636/1.945 m are obtained for along-track component, respectively. C02 had a worse along-track accuracy than C06/C11. As for the cross-track direction, the six-year time series were all stable with RMSs of 1.390/1.281/0.830 m, respectively. The clock precision of C02/C06/C11 was 3.493/2.262/1.921 m, respectively.

**Figure 7:**
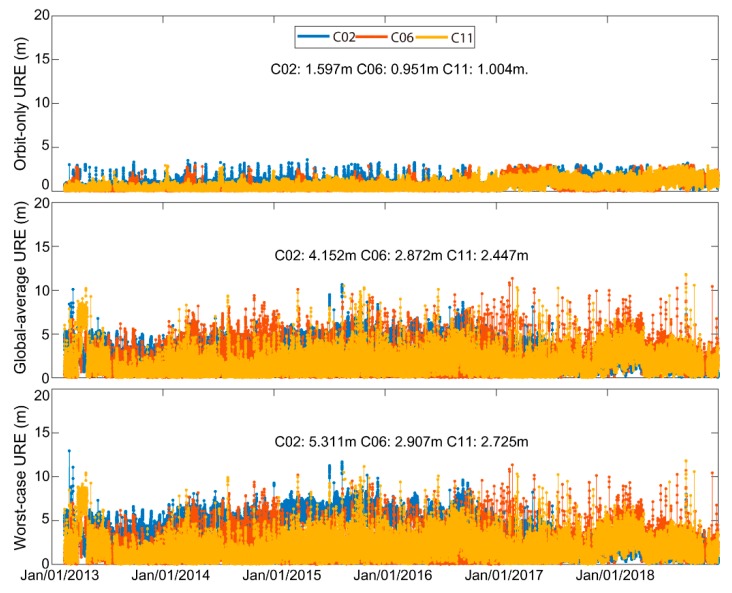
Six-year C02/C03/C11 orbit-only user range errors (UREs), global-average UREs, and worst-case UREs.

Figure 7 shows the six-year time series of three SIS URE criteria for C02/C06/C11. The orbit-only URE RMS of the three satellites is 1.597/0.951/1.004 m, respectively. It is clear that C02 has the worst accuracy. The global-average URE, considering both the orbit and clock errors, owns a larger value than the orbit-only URE. The global-average URE RMSs of C02/C06/C11 are 4.152/2.872/2.447 m, respectively, which is about twice as the orbit-only URE. The instantaneous maximum URE indicator (worst-case URE) owned relatively larger value than the global-average URE, with an RMS of 5.311/2.907/2.725 m for C02/C06/C11, respectively.

**Figure 8:**
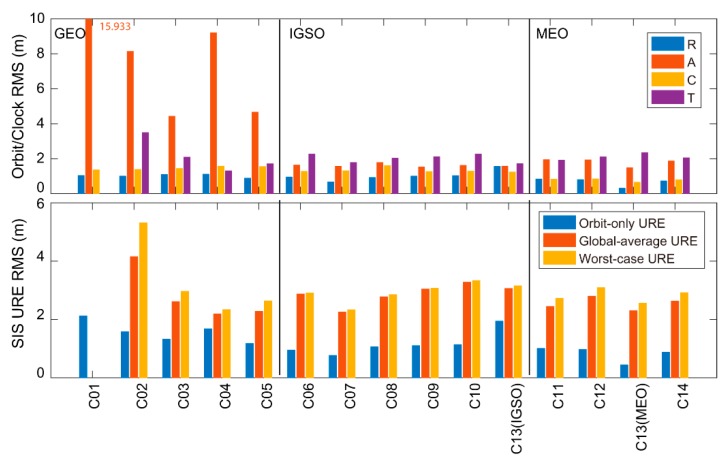
Six-year BDS broadcast navigation message orbit/clock errors and signal-in-space (SIS) UREs RMS.

Figure 8 and Table 4 show the six-year orbit/clock error and SIS URE RMSs for all BDS-2 satellites. Since C01 was selected as the reference satellite for clock assessment, no clock precision statistics were available for C01. Several patterns can be summarized:

(1) Except for C13 (IGSO), radial accuracies for all satellites are the best among three orbit components—better than 1.113 m.
(2) A significant difference between the GEO and IGSO/MEO satellites is reported in the along-track component. A better than 1.945 m RMS is attained for the IGSO/MEO along-track error, whereas a worse than 4.427 m RMS is attained for GEO. C01 is the worst, with a 15.933 m along-track RMS.
(3) With an RMS better than 2.357 m, the clock performance is similar for all satellites except for C02.
(4) Though a large orbit accuracy difference is found in the along-track component among three orbit types, no significant difference is reported for the orbit-only URE. This is because the along-track error is scaled by a factor of 1/54 and 1/127 for MEO and IGSO/GEO, respectively. The orbit-only URE RMS for all satellites is better than 2.122 m.
(5) Better than 3.279 and 3.334 m RMSs are obtained for the BDS-2 global-average URE and worst-case URE, respectively, except for C02. Since the clock error dominates the global-average URE and worst-case URE, C02 owns the worst accuracy on these two criteria, with an RMS of 4.152/5.311 m, respectively, due to the worst clock precision.

All in all, although significantly orbit difference exists between the GEO and IGSO/MEO satellites, the satellite orbit type (GEO/IGSO/MEO) does not impact the orbit-only URE, global-average URE, and worst-case URE.

## 5. Conclusions

Through analyses of MGEX archived BDS-2 broadcast navigation messages from 2013 to 2018, this study reports the six-year BDS-2 broadcast navigation message performance from the perspectives of message availability, satellite anomalies, and SIS UREs. Precise products generated by Wuhan University were collected during the same period (DOY 42, 2013 to DOY 365, 2018) for reference purpose.

Firstly, all BDS-2 satellites provide consecutive availability for six years except for C13, which was launched as an MEO satellite and then switched to an IGSO satellite. The BDS-2 service availability assessment using MGEX navigation files shows a significant difference between GEO/IGSO and MEO. Better than 98.51% navigation message availability is reported for GEO/IGSO, while ~90.03% availability is reported for MEO. Among all available BDS-2 broadcast navigation messages, 651,038 were retained after excluding invalid messages in the preprocessing stage.

Secondly, satellite anomalies were detected based on broadcast navigation messages and precise products. Two indicators in the broadcast navigation messages, the URAI and SV health, were used to detect satellite anomalies. However, our results indicate that the URAI could not reflect any satellite anomalies, while SV health can only partially report satellite anomalies, with 5261 unhealthy messages detected. With a precise orbit/clock as the reference, a total of 20,241 abnormal messages were detected. In these abnormal navigation messages, 18,432 were recognized as orbit anomalies, which is much more frequent than the 2011 clock anomalies.

Finally, 630,797 messages were retained for the six-year BDS-2 nominal SIS UREs assessment. The statistics show that the radial component has the best accuracy for all BDS-2 satellites (except for C13 (IGSO)), with an RMS better than 1.559 m. A significant difference is reported in the along-track component between GEO and IGSO/MEO. A better than 1.945 m RMS is obtained for the IGSO/MEO along-track component, while a >4.427 m RMS is achieved for GEO. However, the difference in the along-track component is not absorbed by the orbit-only URE, since the criterion is not dominated by along-track error. A better than 2.122 m RMS of the orbit-only URE is obtained for all BDS-2 satellites. As for clock performance, except for C02 with a 3.493 m RMS, all satellites achieve a <2.357 m RMS. Since the clock error dominates global-average URE and worst-case URE, the performance of C02 is the worst, with an RMS of 4.152/5.311 m for these two criteria, respectively. For other satellites, <3.279/3.334 m RMSs were obtained for the global-average URE and worst-case URE, respectively. Though a significant orbit difference exists between the GEO and IGSO/MEO satellites, the orbit difference does not affect the SIS UREs.

The outcomes of the six-year BDS-2 broadcast navigation message performance assessment in this study will undoubtedly bring users a clearer understanding of BDS-2 broadcast navigation messages and, thus, more widespread BDS-2 PNT applications.

## Figures and Tables

**Table 1:** BDS-2 anomalies detected by the URAI and SV health.

Orbit Type	PRN #	# of Messages	# of URAI Detected Anomalies	# of SV Health Detected Anomalies	Anomaly Ratio (%)
**GEO**	C01	48,226	0	17	0.04
	C02	48,234	0	46	0.10
	C03	47,240	0	50	0.11
	C04	49,143	0	48	0.10
	C05	48,686	0	58	0.12
	**Total**	**241,529**	**0**	**219**	**0.09**
**IGSO**	C06	49,599	0	181	0.36
	C07	49,654	0	110	0.22
	C08	49,878	0	365	0.73
	C09	49,744	0	71	0.14
	C10	49,836	0	85	0.17
	C13 (IGSO)	19,573	0	12	0.06
	**Total**	**268,284**	**0**	**824**	**0.31**
**MEO**	C11	45,344	0	1305	2.88
	C12	45,610	0	517	1.13
	C13 (MEO)	4949	0	1573	31.78
	C14	45,322	0	823	1.82
	**Total** **(without C13)**	**136,276**	**0**	**2645**	**1.94**

**Table 2:** BDS-2 anomalies detected by precise orbit/clock products.

Orbit Type	PRN #	# of Messages	# of Orbit Anomalies	# of Clock Anomalies	# of Orbit/Clock Anomalies	Anomaly Ratio (%)
**GEO**	C01	48,226	1532	65	1575	3.27
	C02	48,234	989	88	1055	2.19
	C03	47,240	1135	112	1208	2.56
	C04	49,143	1490	65	1554	3.16
	C05	48,686	1319	81	1399	2.87
	**Total**	**241,529**	**6465**	**411**	**6791**	**2.81**
**IGSO**	C06	49,599	1697	74	1769	3.57
	C07	49,654	1376	63	1429	2.88
	C08	49,878	1461	166	1625	3.26
	C09	49,744	1135	31	1166	2.34
	C10	49,836	1291	41	1332	2.67
	C13 (IGSO)	19,573	483	24	506	2.58
	**Total**	**268,284**	**7443**	**399**	**7827**	**2.92**
**MEO**	C11	45,344	1607	639	2215	4.88
	C12	45,610	1401	232	1621	3.55
	C13 (MEO)	4949	78	102	154	3.11
	C14	45,322	1438	228	1633	3.60
	**Total** **(without C13)**	**136,276**	**4446**	**1099**	**5469**	**4.01**

**Table 3:** Six-year BDS anomaly detection result using the URAI, SV health and the precise product.

	URAI Detected	SV Health Detected	Precise Product Detected
Counts	0	5261	20,241
Ratio	0.00%	0.81%	3.11%

**Table 4:** Statistics of six-year BDS broadcast navigation message orbit/clock errors and SIS UREs RMS.

Orbit Type	PRN #	RMS (m)						
		R	A	C	T	Orbit-Only URE	Global-Average URE	Worst-Case URE
**GEO**	C01	1.040	15.933	1.364	-	2.122	-	-
	C02	1.006	8.135	1.390	3.493	1.579	4.152	5.311
	C03	1.099	4.427	1.441	2.091	1.323	2.612	2.962
	C04	1.113	9.200	1.577	1.307	1.676	2.191	2.335
	C05	0.889	4.663	1.549	1.713	1.173	2.282	2.635
**IGSO**	C06	0.951	1.636	1.281	2.262	0.951	2.872	2.907
	C07	0.666	1.570	1.309	1.782	0.767	2.248	2.334
	C08	0.927	1.783	1.610	2.034	1.058	2.774	2.850
	C09	1.004	1.529	1.267	2.117	1.098	3.041	3.072
	C10	1.028	1.618	1.292	2.266	1.134	3.279	3.334
	C13(IGSO)	1.559	1.574	1.242	1.720	1.939	3.061	3.152
**MEO**	C11	0.838	1.945	0.830	1.921	1.004	2.447	2.725
	C12	0.802	1.935	0.849	2.102	0.973	2.794	3.093
	C13(MEO)	0.309	1.487	0.659	2.357	0.440	2.299	2.558
	C14	0.732	1.867	0.798	2.053	0.879	2.627	2.916
